# Field Evolved Resistance in *Helicoverpa armigera* (Lepidoptera: Noctuidae) to *Bacillus thuringiensis* Toxin Cry1Ac in Pakistan

**DOI:** 10.1371/journal.pone.0047309

**Published:** 2012-10-15

**Authors:** Anwaar H. K. Alvi, Ali H. Sayyed, Muhammad Naeem, Muhammad Ali

**Affiliations:** 1 Department of Entomology, PMAS Arid Agriculture University, Rawalpindi, Pakistan; 2 Institute of Molecular Biology & Biotechnology, Bahauddin Zakariya University, Multan, Pakistan; Universidad Nacional Autonoma de Mexico, Instituto de Biotecnologia, Mexico

## Abstract

*Helicoverpa armigera* (Hübner) *is* one of the most destructive pests of several field and vegetable crops, with indiscriminate use of insecticides contributing to multiple instances of resistance. In the present study we assessed whether *H. armigera* had developed resistance to Bt cotton and compared the results with several conventional insecticides. Furthermore, the genetics of resistance was also investigated to determine the inheritance to Cry1Ac resistance. To investigate the development of resistance to Bt cotton, and selected foliar insecticides, *H. armigera* populations were sampled in 2010 and 2011 in several cotton production regions in Pakistan. The resistance ratios (RR) for Cry1Ac, chlorpyrifos, profenofos, cypermethrin, spinosad, indoxacarb, abamectin and deltamethrin were 580-fold, 320-, 1110-, 1950-, 200-, 380, 690, and 40-fold, respectively, compared with the laboratory susceptible (Lab-PK) population. Selection of the field collected population with Cry1Ac in 2010 for five generations increased RR to 5440-fold. The selection also increased RR for deltamethrin, chlorpyrifos, profenofos, cypermethrin, spinosad, indoxacarb, abamectin to 125-folds, 650-, 2840-, 9830-, 370-, 3090-, 1330-fold. The estimated LC_50s_ for reciprocal crosses were 105 µg/ml (Cry1Ac-SEL female × Lab-PK male) and 81 g µg/ml (Lab-PK female × Cry1Ac-SEL male) suggesting that the resistance to Cry1Ac was autosomal; the degree of dominance (D_LC_) was 0.60 and 0.57 respectively. Mixing of enzyme inhibitors significantly decreased resistance to Cry1Ac suggesting that the resistance to Cry1Ac and other insecticides tested in the present study was primarily metabolic. Resistance to Cry1Ac was probably due to a single but unstable factor suggesting that crop rotation with non-Bt cotton or other crops could reduce the selection pressure for *H. armigera* and improve the sustainability of Bt cotton.

## Introduction

The cotton bollworm, *Helicoverpa armigera* (Hübner) (Noctuidae), is one of the most damaging and cosmopolitan pests causing significant economic loss to a wide range of field and vegetable crops [1]. Due to its wider host range, high fecundity, multiple generations, migratory behavior and insecticide resistance, it has become a much more difficult pest to manage [2]. The frequent and indiscriminate use of insecticides has resulted in the development of resistance in many insect populations [3], [4]. Resistance to a wide range of insecticides in *H. armigera* has been reported world-wide, including Pakistan [2]. Moderate to high levels of resistance to pyrethroid and organo-phosphate insecticides was previously reported in a field population of *H. armigera* [5].

Selection for resistance to insecticides in the laboratory is an example of natural selection, and the factors responsible for the cause and increase of resistance-associated mutations are of applied importance. Most of the targets for insecticides are important receptors or enzymes in the insect nervous system, where, poisoning leads to rapid paralysis and insect death. The number of genes selected for resistance depends on whether selection acts within or outside of the phenotypic distribution of the susceptible population [6]. Selection from within this distribution selects preferentially for polygenic resistance, by combining common pre-existing resistance factors that have a minor effect, such as body size and have a major effect. To overcome resistance and to sustain cotton production, the agrochemical industry has recently introduced new chemistries with novel modes of action [7]. Importantly the introduction of *Bacillus thuringiensis* (*Bt*) cotton has revolutionized cotton production globally. However, Bt cotton may not be the optimal solution for all pest problems, owing to the highly specific mode of action of individual Cry toxins against target pests [8]. Without proper resistance management strategies the life expectancy of this technology is likely to be short lived. The High-Dose, Refuge (HDR) strategy, which requires farmers to grow non *Bt* crops near *Bt* crops, can be deployed as either a separate refuge, in which 20% of the field is planted with non-transgenic plants that can be treated with a non-*Bt* foliar insecticide, or as a 4% refuge of non-transgenic plants that are left untreated.

Strategies for delaying insect pest resistance to cotton and maize expressing *Bt* Cry toxins were implemented from the introduction of these transgenes in 1996 and have so far proven to be effective in the US and other developed countries. In contrast, *Pectinophora gossypiella* from India and China has been shown to develop resistance to *Bt* transgenic cotton [9], [10]. We were therefore interested in examining a similar trend in Pakistan, in *H. armigera,* as most of the growers in Pakistan do not follow the HRD strategy. We therefore surveyed the primary cotton growing areas of Pakistan to investigate whether *H. armigera* has developed resistance to the Bt toxin Cry1Ac after exposure to Bt cotton in the field. We further examined the number genes involved in resistance to Cry1Ac in field collected *H. armigera* and mechanisms involved in resistance to Cry1Ac and chemical insecticides.

## Results

### Toxicity of Insecticides to a Laboratory Susceptible Population and Field Population

Toxicity of chlorpyrifos, profenofos Cry1Ac, indoxacarb and deltamethrin to the laboratory susceptible, Lab-PK was similar (overlapping of 95% FL; P>0.05), but higher for cypermethrin and abacmectin (Table 1). In contrast, the toxicity of spinosad was significantly lower (non-overlapping of 95% FL; P<0.05) than cypermethrin and abamectin but was similar to other insecticides tested (Table 1). The slopes for all insecticides tested against Lab-PK were similar, but more shallow indicating that the response in the laboratory susceptible population to tested insecticides was heterogenous.

**Table 1:** Toxicity of various insecticides to laboratory susceptible (Lab-PK) and field collected populations of *H. armigera.*

Population	Insecticides	LC_50_ (95% FL)(µg/ml)	Slope ± SE	RR^1^	DR^2^	n^3^
Susceptible	Cry1Ac	0.58 (0.28–1.20)	1.07±0.16	–	–	240
Susceptible	Chlorpyrifos	0.46 (0.18–0.96)	1.03±0.17	–	–	240
Susceptible	Profenofos	0.50 (0.20–1.03)	1.06±0.17	–	–	240
Susceptible	Cypermethrin	0.26 (0.12–0.50)	1.20±0.17	–	–	240
Susceptible	Spinosad	1.45 (0.69–2.79)	1.13±0.18	–	–	240
Susceptible	Indoxacarb	0.90 (0.48–1.71)	1.27±0.17	–	–	240
Susceptible	Abamectin	0.23 (0.08–0.54)	0.85±0.14	–	–	240
Susceptible	Deltamethrin	0.42 (0.17–0.78)	1.27±0.22	–	–	240
Field	Cry1Ac	335.7 (244.2–477.6)	2.43±0.31	579	–	240
Field	Chlorpyrifos	148.7 (106.3–217.0)	2.29±0.27	323	–	240
Field	Profenofos	557.4 (420.3–734.5)	3.20±0.46	1115	–	240
Field	Cypermethrin	506.8 (387.7–680.7)	3.28±0.44	1949	–	240
Field	Spinosad	284.7 (180.0–484.7)	1.74±0.30	196	–	240
Field	Indoxacarb	341.2 (260.8–459.7)	3.08±0.34	379	–	240
Field	Abamectin	159.2 (121.3–208.8)	3.15±0.53	692	–	240
Field	Deltamethrin	16.02 (12.74–20.32)	3.49±0.45	38		240
Field-UNSEL (G_6_)^4^	Cry1Ac	19.65 (11.56–33.04)	1.50±0.22	34	−0.21	240
Field-UNSEL (G_6_)	Chlorpyrifos	11.75 (7.96–17.83)	2.04±0.25	26	−0.18	240
Field-UNSEL (G_6_)	Profenofos	18.48 (12.30–27.83)	2.05±0.25	37	−0.25	240
Field-UNSEL (G_6_)	Cypermethrin	20.07 (12.71–31.48)	1.83±0.24	77	−0.23	240
Field-UNSEL (G_6_)	Spinosad	9.22 (5.76–14.81)	1.66±0.23	6	−0.25	240
Field-UNSEL (G_6_)	Indoxacarb	8.07 (4.04–14.37)	1.29±0.20	9	−0.27	240
Field-UNSEL (G_6_)	Abamectin	9.27 (5.88–14.79)	1.77±0.24	40	−0.21	240
Field-UNSEL (G_6_)	Deltamethrin	1.76 (1.14–2.61)	1.90±0.30	4	−0.16	240

1 Resistance ratio = LC_50_ of field and unselected (UNSEL) population÷LC_50_ of Lab-PK.

2 Decline in resistance = Log_10_ (initial LC_50_–final LC_50_)÷number of generation the population of unexposed to toxicant.

3 The number of larvae exposed to toxins in bioassays including controls.

4 Generations.

The toxicity of all insecticides tested against a field collected population was significantly lower (P<0.05) at G1 compared with Lab PK. The resistance ratios for chlorpyrifos, profenofos, Cry1Ac, cypermethrin, spinosad, indoxacarb, abamectin and deltamethrin were 320-fold, 1110-, 580-, 1950-, 200-, 380, 690, and 40-fold respectively (Table 1). The slopes of the regression lines for the insecticides tested against field population at G1 were significantly steeper for chlorpyrifos, profenofos, Cry1Ac, cypermethrin, indoxacarb, abamectin and deltamethrin compared with Lab-PK, suggesting a homogenous response in the field collected population to these insecticides. The slope of spinosad however was more shallow than other insecticides but it was similar to Lab-PK (Table 1).

**Figure 1:**
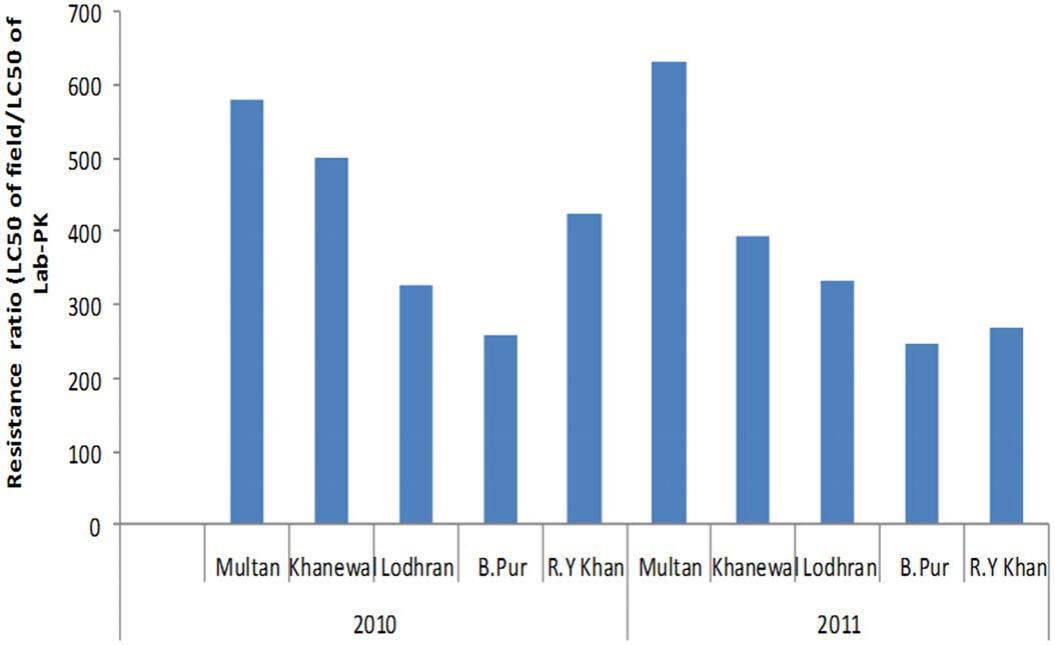
Effect of Cry1Ac on development of resistance in *H. armigera* collected from different areas of Pakistan.

The response of *H. armigera* to Cry1Ac collected from various locations was similar; however the highest resistance ratio was obtained for the population collected from Multan (Fig. 1).

### Response to Selection and Cross-resistance in Cry1Ac-SEL Population

Mortality at different selection doses of 300, 500 and 1000 µg AI mL−1, determined after 7 days exposure to Cry1Ac were 59, 35, 15 and 40% respectively. Selection of the field population with Cry1Ac from G1 to G5 increased the resistance ratio (RR) to 160-fold for Cry1Ac compared with the Unselected field population. However, when it was compared with Lab-PK, the RR increased from 580-fold to 5440-fold (just five generations of selection). Similarly, selection with Cry1Ac also increased RR for deltamethrin, chlorpyrifos, profenofos, cypermethrin, spinosad, indoxacarb, abamectin to 125-fold, 650-, 2840-, 9830-, 370-, 3090- and 1330-fold, compared with Lab-PK (Table 2).

**Table 2:** Cross-resistance and instability pattern in a Cry1Ac-selected (Cry1Ac-SEL) population of *H. armigera*.

Population	Insecticides	LC_50_ (95% FL)(µg/ml)	Slope ± SE	RR^1^	RR^2^	DR^3^	n^4^
Cry1Ac-SEL (G_6_)^5^	Cry1Ac	3154 (2548–4000)	3.65±0.47	5438	160	–	240
Cry1Ac-SEL (G_6_)	Chlorpyrifos	1220 (957.3–1585)	3.28±0.39	2652	104	–	240
Cry1Ac-SEL (G_6_)	Profenofos	1421 (998.8–2022)	2.31±0.28	2842	77	–	240
Cry1Ac-SEL (G_6_)	Cypermethrin	2557 (2093–3164)	4.06±0.50	9834	127	–	240
Cry1Ac-SEL (G_6_)	Spinosad	533.3 (322.2–836.6)	1.88±0.30	368	58	–	240
Cry1Ac-SEL (G_6_)	Indoxacarb	2779 (2293–3424)	4.24±0.51	3088	344	–	240
Cry1Ac-SEL (G_6_)	Abamectin	306.4 (222.1–430.4)	2.52±0.31	1332	33	–	240
Cry1Ac-SEL (G_6_)	Deltamethrin	52.28(36.71–89.46)	2.79±0.41	125	18	–	240
Cry1Ac-UNSEL(G_10_)	Cry1Ac	199.2 (141.6–275.7)	2.36±0.35	343	10	−0.30	240
Cry1Ac-UNSEL(G_10_)	Chlorpyrifos	93.20 (64.45–135.9)	2.17±0.28	203	8	−0.28	240
Cry1Ac-UNSEL(G_10_)	Profenofos	98.61 (65.18–147.4)	2.03±0.25	197	5	−0.29	240
Cry1Ac-UNSEL(G_10_)	Cypermethrin	204.6 (153.5–268.9)	2.96±0.40	787	10	−0.27	240
Cry1Ac-UNSEL(G_10_)	Spinosad	69.11 (44.33–105.4)	1.81±0.27	48	7	−0.22	240
Cry1Ac-UNSEL(G_10_)	Indoxacarb	111.8 (70.27–162.5)	1.99±0.32	124	14	−0.35	240
Cry1Ac-UNSEL(G_10_)	Abamectin	50.38 (34.99–71.88)	2.26±0.31	219	5	−0.20	240
Cry1Ac-UNSEL(G_10_)	Deltamethrin	10.04 (7.85–12.89)	3.59±0.44	24			

1 Resistance ratio = LC_50_ of Cry1Ac-SEL and unselected (UNSEL) populations÷LC_50_ of Lab-PK.

2 Resistance ratio = LC_50_ of Cry1Ac-SEL and Cry1Ac unselected populations÷LC_50_ of UNSEL (Table 1).

3 Decline in resistance = Log_10_ (initial LC_50_– final LC_50_)÷number of generation the population of unexposed to toxicant.

4 The number of larvae exposed to toxins in bioassays including controls.

5 Generations.

Selection with Cry1Ac also increased the slope for the probit line for the Cry1Ac-SEL population compared with the field population at G1. The slope for the insecticides tested against Cry1Ac-SEL also increased significantly indicating increase in homogeneity in the selected population.

**Figure 2:**
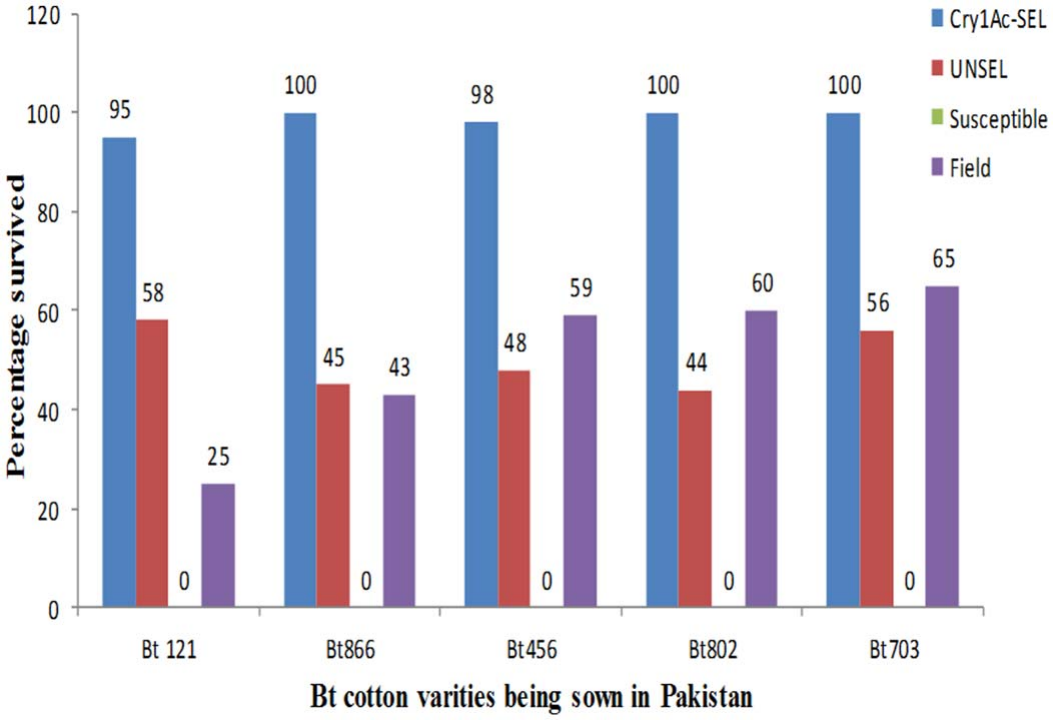
Survival of Cry1Ac-SEL, UNSEL and Lab-PK on different Bt cotton varieties being sown in different parts of Pakistan.

The bioassays on various *Bt* cotton varieties revealed that the survival of Cry1Ac-SEL population was almost 100% on all varieties which was significantly higher than any other strain tested (Fig. 2). In contrast, 100% mortality was obtained with the Lab-PK population while the UNSEL and field population response was similar among varieties (Fig. 2).

### Reversion of Resistance to Cry1Ac and Other Insecticides in Field and Cry1Ac-SEL Populations

In order to investigate the stability of resistance to Cry1Ac and other insecticides in field populations and Cry1Ac-SEL, Cry1AC-SEL was maintained for 6 generations without exposure to insecticides. Bioassays of field populations at G_5_ showed a significant reduction (P<0.05) in resistance ratio with a reversion rate of −0.21. Similarly, rearing field population without exposure to insecticides also reduced ratios for chlorpyrifos, profenofos, cypermethrin, spinosad, indoxacarb and abamectin (Table 1). The reversion rate of resistance to deltamethrin in the field population was the least (−0.16) while it was the highest for indoxacarb (−0.27; Table 1).

The Cry1Ac-SEL population was also monitored for reversion of resistance to deltamethrin and other insecticides for four generations (G_7_–G_10_). Bioassays carried out at G_11_ indicated that resistance to Cry1Ac declined significantly (P<0.05) in four generations, near the level of field evolved resistance. Likewise, resistance to chlorpyrifos, profenofos, cypermethrin, spinosad, indoxacar**,** abamectin and deltamethrin was also reduced significantly from G6 to G10. The rate of decline of resistance in Cry1Ac-SEL population was similar to the reversion rate for the field selected population.

The parameter R is used to estimate response to selection (Falconer, 1989) which can also be applied to determine the number of generations required to a 10-fold change in resistance. The inverse of R is the number of generations required for a 10-fold change in LC_50_. The R value for Cry1Ac is 0.21 suggesting that only five generations are required to increase 10-fold resistance to Cry1Ac.

### Inheritance of Cry1Ac Resistance

The LC_50_ of Cry1Ac for Cry1Ac-SEL population was over 3000-fold which was significantly higher than LC_50_ of Cry1Ac for Lab-PK. Estimated LC_50_ for F_1_ female progeny from reciprocal crosses were 105 µg/ml (Cry1Ac-SEL female×Lab-PK male) and 81g µg/ml (Lab-PK female×Cry1Ac-SEL male). The LC_50_ values and mean slopes for the concentration mortality line did not differ significantly between F_1_ progenies of the reciprocal crosses between Lab-PK and Cry1Ac-SEL suggesting that the inheritance of resistance to Cry1Ac was autosomal; neither maternal effects nor sex linkages were evident (Table 3). The degree of dominance (D_LC_) for the reciprocal crosses was 0.60 for F1 (Cry1Ac-SEL female×Lab-PK male) and 0.57 for F1 (Lab-PK female×Cry1Ac-SEL male), indicating incomplete dominance of resistance to Cry1Ac in Cry1Ac-SEL population.

**Table 3:** Inheritance of resistance to Cry1Ac in a Cry1Ac-SEL population of *H. armigera.*

population	LC_50_ (95% FL)(µg/ml)	Slope ± SE	D_LC_ 1
Cry1Ac-SEL (G_6_)^2^	3154 (2548–4000)	3.65±0.47	–
Lab-PK	0.58 (0.28–1.20)	1.07±0.16	–
Cry1Ac-SEL♀ × Lab-PK ♂	105.5(70.69–163.0)	1.76±0.21	0.60
Lab-PK ♀ × Cry1Ac-SEL♂	81.21(52.19–129.3)	1.56±0.20	0.57
F_1_ × Cry1Ac-SEL	2.68(1.86–4.10)	0.98±0.23	–

1 Degree of dominance at LC_50_, which was calculated as described previously [24].

2 Generations.

Pooled F_1_ progeny were backcrossed to the Lab-PK colony, resulting in a slope of 0.98; this is similar to Lab-PK but about 4-fold less than the estimated slope for Cry1Ac-SEL, about 2-fold less than F1 progeny indicate decreased genetic variance in the backcrossed progeny compared with F1 progeny. The decreased genetic variance indicates the number of loci with major effect on resistance to Cry1Ac was very low. Similarly the direct test for a monogenic mode of inheritance of resistance, which is based on the goodness of fit *χ* ^2^ between the F_1,_ backcross and the Lab-PK expected values, calculated as described by Sokal and Rohlf [11] also suggest involvement of one locus with the resistance. As the monogenic model showed insignificant deviation (*P*>0.05) between observed and expected mortality at seven (Table 3). Likewise, calculation of the minimum number of independently segregating loci with equal and additive contributions to resistance had given an estimate of 0.54, which also supported the conclusion that resistance was conferred by a single locus.

### Resistance to Insecticides is Inhibited by Synergists

The synergistic effects of PBO and DEF on Cry1Ac and other insecticides were determined in Lab-PK and Cry1Ac-SEL populations of *H. armigera*. The monooxygenase specific inhibitor PBO showed 273-fold synergism to Cry1Ac in the Cry1Ac-SEL population at G6; however, no synergism was observed for Lab-PK (Table 4). Only a 2-fold level of resistance remained after the application of PBO and Cry1Ac together, suggesting that the major mechanism was associated with mono-oxygenases or esterases since PBO has also been shown to inhibit the activity of esterases [12]. When the esterase specific inhibitor DEF was used, a high level of synergism (73-fold) was observed against the Cry1Ac-SEL population but no effect of DEF was detected in Lab-PK (Table 4). The occurrence of synergism for both inhibitors suggests that enhanced activities of esterases, or probably mono-oxygenases, are involved in resistance to Cry1Ac in *H. armigera.*

**Table 4:** Susceptibility of Cry1Ac-selected (Cry-SEL) populations of *H. armigera* to Cry1Ac and other insecticides tested in the presence or absence of a PBO or DEF.

Population	Treatment	LC_50_ (95% FL)	Slope ± SE	RR^1^	SR^2^
Lab-PK	Cry1Ac	0.58 (0.28–1.20)	1.07±0.16	–	–
	Cry1Ac+ PBO	0.50 (0.39–1.50)	3.09±0.46	–	1
	Cry1Ac+ DEF	0.48(0.12–1.34)	1.44±0.23	–	1
	Profenofos	0.50 (0.20–1.03)	1.06±0.17	–	–
	Profenofos+ PBO	0.98(0.52–1.60)	1.58±0.24	–	1
	Profenofos+ DEF	0.44(0.15–0.95)	1.01±0.17	–	1
	Indoxacarb	0.90 (0.48–1.71)	1.27±0.17	–	–
	Indoxacarb+ PBO	1.05 (0.62–3.10)	3.54±0.49	–	1
	Indoxacarb+ DEF	1.29 (0.81–2.44)	1.79±0.27	–	1
	Deltamethrin	0.42 (0.17–0.78)	1.27±0.22	–	–
	Deltamethrin + PBO	0.14 (0.08–0.43)	1.56±0.23	–	
	Deltamethrin+ DEF	0.09 (0.03–0.23)	1,23±0.13	–	
Cry1Ac-SEL	Cry1Ac	315.4(254.8–540.3)	1.65±0.37	544	–
	Cry1Ac+ PBO	1.15(0.56–2.44)	1.07±0.15	2	274
	Cry1Ac+ DEF	4.31(2.80–6.66)	1.82±0.27	9	73
	Profenofos	421(221.8–602.7)	2.17±0.29	842	–
	Profenofos+ PBO	1.27(0.76–2.06)	1.79±0.27	1	331
	Profenofos+ DEF	4.85(3.44–6.57)	2.82±0.45	11	87
	Indoxacarb	277.9(122.2–436.7)	2.24±0.41	309	–
	Indoxacarb+ PBO	1.25 (0.70–2.27)	1.40±0.18	1	222
	Indoxacarb+ DEF	4.54 (3.14–6.52)	2.26±0.31	4	61
	Deltamethrin	52.3 (36.7–89.5)	2.79±0.41	125	–
	Deltamethrin + PBO	5.12 (1.32–15.6)	1.93±0.23	37	10
	Deltamethrin+ DEF	3.25 (1.11–11.3)	2,12±0.14	36	16

1 Resistance ratio was LC_50_ of Cry1Ac or insecticides for Cry1Ac-SEL÷LC_50_ of Cry1Ac or insecticides for Lab-PK.

2 The synergism ratio (SR) was calculated from LC_50_ of Cry1Ac or other insecticides tested÷LC_50_ of Cry1Ac+inhibitor or insecticides+inhibitor.

Similarly, when insecticides like profenofos and indoxacarb were used in a mixture with either PBO or DEF against Cry1Ac-SEL population, a high level of synergism was observed but there was no effect of inhibitors on Cry1Ac toxicity against Lab-PK. The most intriguing observation was synergism of PBO or DEF with Cry1Ac against Cry1Ac-SEL population (Table 4).

## Discussion

Our data suggest that *H. armigera* has developed resistance to Cry1Ac in the field in Pakistan. In Pakistan illegal planting of *Bt* cotton has occurred since 1999, without following the HRD strategy. Previous studies from India and China had reported field evolved resistance to Cry1Ac in *Pectinophora gossypiella* [9], [13]. In the present study, our data show a high level of resistance, not only to Cry1Ac, but also to conventional insecticides such as pyrethroids and organophosphates. To confirm whether the resistance to Cry1Ac and conventional insecticides was associated with the same mechanism of resistance, selection experiments were performed in the laboratory with Cry1Ac on a field collected population. After six generations of selection, resistance to Cry1Ac increased significantly (non-overlapping of 95% FL). Cross-resistance patterns in Cry1Ac-SEL could result from enzymes such as metabolic enzymes [8], [12] and mutation at an insecticidal target site [3]. The high level of resistance shown by the Cry1Ac-SEL population suggests either a common mechanism affecting these insecticides or genetically linked independent mechanisms for Bt toxin Cry1Ac and deltamethrin. The findings of the present study are similar to previously reported results of *Plutella xylostella* resistance to deltamethrin, which showed a high level of reciprocal cross-resisance to Cry1Ac [8]. Similarly, our data also suggest that the resistance to Cry1Ac in the selected population was due to involvement of metabolic enzymes as was previously shown for *P. xylostella* [8] or *H. armigera* [12]. The metabolic enzymes have several isoenzymes that can act on different insecticides; if an insecticide selects some isoenzymes that can affect different insecticides then cross-resistance is possible [14]. When PBO, mono-oygenase or esterases inhibitor or DEF esterases inhibitors were used in combination with Cry1Ac or deltamethrin, the resistance to both toxicants was reduced significantly suggesting that the major mechanism of resistance to Cry1Ac or deltamethrin was associated with esterases. We also carried out bioassays with profenofos and indoxacarb in the presence of PBO or DEF and the data suggest that the resistance to the insecticides was associated with metabolic enzyme, esterases. The Cry1Ac-SEL population was derived from a field population which was collected from an area where Bt cotton has been grown since 1999. However pyrethroids and neonicotinoids are also being sprayed to control sucking insect pests such as *Bemisia tabaci,* suggesting that *H. armigera* in Pakistan is not only exposed to Bt toxin Cry1Ac but also to conventional insecticides. The common mechanism of resistance to several insecticides as shown by Cry1Ac-SEL, is literally a result of field exposure. Although laboratory experiments cannot completely reflect field conditions, our results are consistent with the fact that the rapid development of resistance to Cry1Ac and deltamethrin observed in field populations was due to exposure to insecticides in the field.

A field collected population was also selected for susceptibility to different insecticides. It was found that after one year of selection in the laboratory for 14 generations, the susceptibility to insecticides increased significantly. The resulting Lab-PK was significantly more susceptible to insecticides than another laboratory population of *H. armigera* from Pakistan with identical bioassay systems to the present studies [5]. The most probable reason for high susceptibility in the Lab-PK population was due to its collection from a non-cotton growing (Islamabad). Resistance alleles, if present at the time of collection, were likely lost due to rearing the pest without exposure to insecticides.

Data obtained on the stability of the mechanism of resistance in the selected or the field collected population suggest instability of resistance as the LC_50_ decreased significantly in the absence of selection. Rapid reversion of resistance in the Cry1Ac-SEL population and field collected population suggest that high fitness costs may be associated with resistance. The decline in resistance could also be due to the presence of heterozygotes in the selected population. A high level of resistance to conventional insecticides has been reported to decline rapidly in populations selected in the laboratory or in the field [15]. The rapid decline in resistance in the Cry1Ac-SEL population indicates that if the toxin is removed from the field, the resistance could also decline quickly. However, this is unlikely in Pakistan as farmers are maintaining Bt cotton throughout the year as a ratoon crop. This is a serious concern and will likely increase the selection pressure, allowing resistance to rapidly spread to other areas of Pakistan.

Reciprocal crosses between resistant and susceptible populations can provide information on dominance of resistance genes, sex linkage and the number of genes involved in resistance to insecticides. The results of these crosses between Cry1Ac-SEL and Lab-PK showed no significant difference in LC50s of reciprocal crosses, suggesting that the resistance was autosomal and no sex linkage was observed. Like resistance to deltamethrin and indoxacarb in *Spodoptera litura,* and Cry1Ac, deltamethrin and spinosad resistance in *P. xylostella* from Pakistan, resistance in *H. armigera* was incompletely dominant. The resemblance of genetics of resistance among various compounds could be due to similarity in selection protocols as the selection is carried out with the aim of having about 50% survival of exposed larvae, which could lead to an incompletely dominant mode of inheritance [14]. However the mode of inheritance at a given concentration of insecticide also depends upon the life stage of an insect especially first instars are generally more susceptible than later instars [8], [16]. Although we have not carried out assays at first instar but our observations suggest that F_1_ progeny of Cry1Ac-SEL was more susceptible at neonate stage than the second instar (the stage which was tested in bioassays). Resistance to insecticides is generally monogenic [17]. The backcrossing of F_1_ progeny to parents usually support the estimate the number of genes involved in resistance [18] and the data of the present study suggest that resistance to Cry1Ac in the Cry1Ac-SEL population is controlled by single gene. The minimum number of effective factors estimated in the present study are less than 1 (nE <1), which also suggests that resistance is controlled primarily by one locus [19].

Stability of resistance and cross-resistance of Cry1Ac to deltamethrin suggest that common resistance mechanism was linked with the failure of two distant pest management control agents against *H. armigera* from Pakistan. Our data show a significant survival of the Cry1Ac-SEL, UNSEL and field collected *H. armigera* on Bt cotton (Fig. 2) suggesting that even small decreases in susceptibility to Cry1Ac could reduce the efficacy of Bt cotton in the field. Based on the field data described above and our bioassay results, we hypothesize that the magnitude of resistance documented here reduces the efficacy of Cry1Ac producing Bt cotton against *H, armigera* in the field. The results on *Pectinophora gossypiella* resistance to Cry1Ac from India and China suggest that the refuge strategy has helped to delay resistance [9]. In contrast our study suggests that field collected population of *H. armigera* survived on Bt cotton. The most probable reasons for the survival are that in Pakistan cotton growers are using Bt cotton varieties which do not have sufficiently high expression of Cry1Ac. Similarly, small land-holding farmers are not using the refuge of non Bt cotton to increase the population of hybrid progeny. Recently due to high cotton prices in the market, growers in Pakistan are keeping the cotton plants through out the year as a ratoon crop which is also exposing *H. armigera* a year around to Bt toxin and thus increasing the chances of resistance development.

Most of the Bt cotton varieties being planted in Pakistan were locally developed. Varieties developed by Monsanto were not approved to grow in Pakistan. While in other countries such as Australia and the US key conditions of the HDR strategy are being met and susceptibility to Cry1Ac has not decreased substantially in the target pests, despite a relatively high initial frequency of resistance [12], [13]. The most important option to counter resistance in *H. armigera* is to switch to Bt cotton expressing two toxins, Cry2Ab and Cry1Ac [13]. However, for long-term sustainable IRM, cotton with two or more toxins other than Cry1Ac would be better for countering resistance to Cry1Ac [13]. A second option, which is very unlikely in Pakistani agriculture, is to increase plantings of non-Bt cotton. Finally using other control tactics, such as cultural practices in combination with Bt cotton, could be another option to suppress resistance to Bt and conventional insecticides.

## Materials and Methods

### Insects

**Figure 3:**
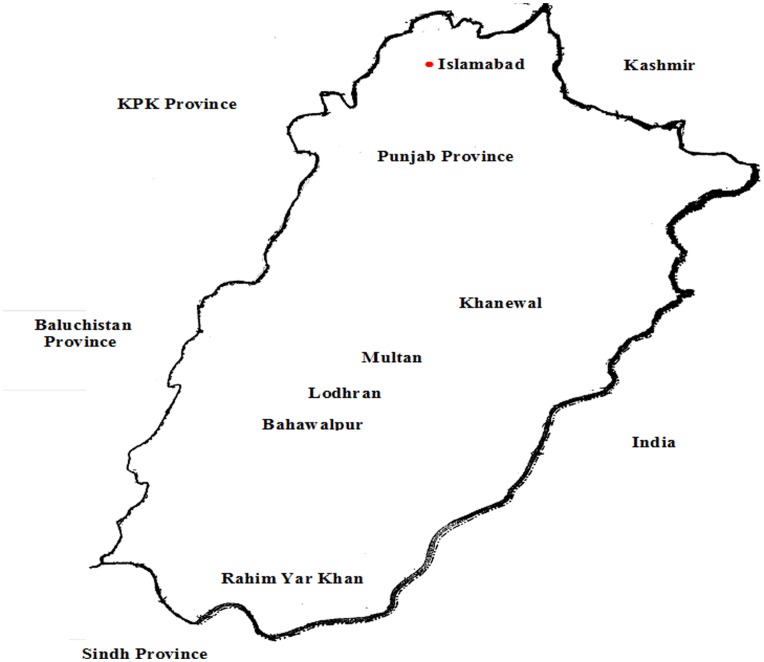
Sampling site for *H. armigera* from Punjab province, Pakistan.

*Helicoverpa armigera* larvae were collected from non Bt cotton fields, as the treatment regimes used provide a greater chance for the generation of resistance than the regimes used in vegetables. By pest scouting of fields from five districts, namely Multan, Khanewal, Lodhran, Bahawalpur and Rahim Yar Khan approximately 400 larvae were collected in 2010. No specific permit was required to collect insects from the field as the fields were privately owned and merely by speaking to private owners, collection was made. Since the collection was not involved endangered species therefore no such permission was required from any concerned authority in Pakistan. The areas are in Punjab Province and under multiple cropping systems, with several cultivated crops such as cotton, maize, sorghum, millet, rice, sugarcane, wheat, potato, vegetables, fodder crops and orchards (Fig. 3). These crops are grown side by side, depending on the season. An insecticide-susceptible population, labeled as Lab-PK, was collected from Multan, Punjab province from non Bt cotton field and selected for susceptibility in the laboratory as described previously [14].

Larvae were reared on semi-synthetic wheatgerm-based diet in the laboratory at 25±2°C and 60–65% relative humidity with a 14∶10 h light : dark photoperiod.4 Diet was replaced after 24 h, and pupae were collected on alternate days. The adults that emerged were kept in Perspex oviposition cages (30×30×30 cm) with two sides sealed with muslin to maintain ventilation and fed on a solution containing sucrose (100 g L^−1^), vitamin solution (20 mL L^−1^) and methyl 4-hydroxybenzoate (2 g L^−1^) presented on a soaked cotton wool ball.

### Insecticides

Commercial formulations of the different insecticides used in bioassays included spinosad (Tracers 24SC, Dow Agro-Sciences, UK), indoxacarb (Stewards15SC, DuPont, Pakistan), abamectin (Agrimec TM 1.8EC, Syngenta, UK), cypermethrin 100 g L−1 EC (Arrivo® 10EC; FM, Philadelphia, PA), deltamethrin 105 g L−1 EC (Decis Super® 10.5EC; Bayer Crop Science, France), Profenofos 500 g L−1 EC (Curacron® 50EC; Syngenta Crop Protection, Switzerland), chlorpyrifos 400 g L−1 EC (Lorsban® 40EC; Dow AgroSciences, UK) and Cry1Ac. The source of Cry1Ac was a lyophilized (freeze-dried) formulation of MVP II containing ≈20% Cry1Ac protoxin of *B. thuringiensis* variety *kurstaki* encapsulated by transgenic *Pseudomonas fluorescens* Migula (Mycogen Corporation, San Diego, CA). It was stored at −80°C until used and before use it was allowed to warm at room temperature. Appropriate amounts of the lyophilized material were weighed for each concentration and suspended in distilled water.

### Bioassays

Second instar *H. armigera* larvae were used for all bioassays, in which the insecticides or Cry1Ac was incorporated into an artificial wheatgerm diet [20]. The Cry1Ac or insecticides were serially diluted with distilled water and then mixed with diet at an appropriate temperature of diet. Toxins incorporated freshly prepared diet was poured into 140-ml plastic Petri-dishes. For controls, distilled water was mixed with the diet. All assays included seven to eight toxin doses (concentrations) each with three to eight replicates and 30 larvae were placed on each replicate. The Petri-dishes were wrapped black paper to avoid cannibalism [21] and incubated at 27°C, 70% RH, and a photoperiod of 14∶10 (L:D) h for 7 d. The *H. armigera* are known to cannibalized however if they are placed in dark place, this behavior can be avoided [21]. Larval mortality and stunting (larvae that failed to molt to third instars) were recorded as response data. Dosage mortality data were analyzed by probit analysis [22].

We used five Bt cotton varieties viz. Bt121, Bt856, Bt456, Bt802 and Bt703, which expressed Cry1Ac to determine survival of field collected, Cry1Ac-SEL, UNSEL and Lab-PK populations on these varieties. These varieties were grown in pots of 45 cm to 30 cm using clay loam soil with farm yard manure as organic fertilizers. The pots were kept in an open field to avoid damage to Cry1Ac as it is an established fact that the expression of toxin declined with the age and also the plants will be less toxic if they are grown in the greenhouse. The five first instar larvae per plant were released on eight weeks old 10 plants of each variety and the larvae were allowed to complete their larval stage. The plants with larvae were placed at 27°C, 70% RH, and a photoperiod of 14∶10 (L:D) h.

### Selection with Cry1Ac

The field collected population was divided into two sub-populations. One population was selected with Cry1Ac (Cry1Ac-SEL) while the second population was left unselected (UNSEL) for five generations. The selection was done using three concentrations of MVPII viz 300, 500 and 1000 µg AI mL^−1^ and about 300 larvae were used in each round of selection. The larvae were exposed to the toxin for seven days and after exposing the larvae to toxin the survived larvae were reared on freshly prepared diet without toxin until they pupated. Mean survival of larvae after exposure to Cry1Ac concentrations over four generations was 35% for Cry1Ac-SEL.

### Effect of Inhibitors on Pesticides Activities

The toxicities of Cry1Ac, profenofos, indoxacarb and deltamethrin to Cry1Ac-SEL, UNSEL, and Lab-PK populations were determined in the presence of two inhibitors, piperonyl butoxide (PBO; Sigma Ltd, UK), an inhibitor of cytochrome P450 monooxygenases (microsomal oxidases) and of esterases, and *S*,*S*,*S*-tri-*n*-butyl phosphorotrithioate (DEF; Sigma Ltd, UK), an esterase-specific inhibitor as described previously [8]. The 10 mg L^−1^ of the inhibitor was added to various concentrations of the pesticides and larvae were exposed as described above. The mortality was recorded after seven days exposure to the pesticides. The synergism ratio (SR) was calculated by dividing the LC_50_ of the population treated with pesticide alone by the LC_50_ of the strain treated with pesticide plus the inhibitor.

### Genetics of Resistance to Cry1Ac

The response of F1 progeny to Cry1Ac was evaluated in mass reciprocal crosses between Cry1Ac-SEL and laboratory susceptible (Lab-PK) populations. To produce F1, mass crosses using 50 adults of each sex provided enough offspring for multiple-concentration testing and calculation of the 50% lethal concentration (LC50). The degree of dominance for LC50 (*D*LC) was calculated as described by Sayyed et al [23] and Bourguet *et al*. [24] Backcrossed offspring were obtained from F1× Lab-PK. This backcross was preferred to F1×Cry1Ac-SEL because the resistance was incompletely dominant and differed more from Lab-PK than from Cry1Ac-SEL.

### Data Analysis

Mortality data were corrected by Abbott’s formula [25] where necessary and analysed by probit analysis [26] using the software POLO-PC [22]. The estimates of LC50 values and their 95% fiducial limits (FL) were obtained by probit analysis using Polo. Because of the inherent variability of bioassays, pair-wise comparisons of LC50 values were made at the 1% significance level where individual 95% FL for two treatments do not overlap [27]. Resistance ratios were determined by dividing the LC50 values of field populations by the LC50 of Lab-PK. Cross-resistance pattern among insecticides was studied with pair-wise correlation co-efficient of LC50 values of the field populations for each insecticide.
